# Decreased expression of the endothelial cell-derived factor EGFL7 in systemic sclerosis: potential contribution to impaired angiogenesis and vasculogenesis

**DOI:** 10.1186/ar4349

**Published:** 2013-10-25

**Authors:** Mirko Manetti, Serena Guiducci, Eloisa Romano, Jérôme Avouac, Irene Rosa, Barbara Ruiz, Gemma Lepri, Silvia Bellando-Randone, Lidia Ibba-Manneschi, Yannick Allanore, Marco Matucci-Cerinic

**Affiliations:** 1 Department of Experimental and Clinical Medicine, Section of Anatomy and Histology, University of Florence, Largo Brambilla 3, 50134 Florence, Italy; 2 Department of Experimental and Clinical Medicine, Section of Internal Medicine and Division of Rheumatology, Azienda Ospedaliero-Universitaria Careggi (AOUC), University of Florence, Villa Monna Tessa, Viale Pieraccini 18, 50139 Florence, Italy; 3 Rheumatology A Department, Paris Descartes University, Sorbonne Paris Cité, Cochin Hospital, Paris, France; 4 Cochin Institute, Paris Descartes University, INSERM U1016 and CNRS UMR8104, Paris, France

## Abstract

**Introduction:**

Microvascular damage and defective angiogenesis and vasculogenesis have a major role in the pathogenesis of systemic sclerosis (SSc). Epidermal growth factor-like domain 7 (EGFL7) is a proangiogenic molecule which is predominantly expressed and secreted by endothelial cells and their progenitors and controls vascular development and integrity. In this study, we investigated the possible involvement of EGFL7 in SSc.

**Methods:**

Serum EGFL7 levels from 60 patients with SSc and 35 age- and sex-matched healthy controls were examined by colorimetric sandwich enzyme-linked immunosorbent assay. The expression of EGFL7 in forearm skin biopsies (n = 16 SSc, n = 10 controls), cultured dermal microvascular endothelial cells (MVECs) (n = 3 SSc, n = 3 controls) and late-outgrowth peripheral blood endothelial progenitor cell (EPC)-derived endothelial cells (n = 15 SSc, n = 8 controls) was investigated by immunofluorescence and Western blotting.

**Results:**

Serum EGFL7 levels were detectable in 68.6% of healthy controls and 45% of SSc cases (*P* < 0.05). Circulating levels of EGFL7 were significantly decreased in SSc patients compared with healthy controls (*P* = 0.01). Serum levels of EGFL7 were significantly lower in both limited cutaneous SSc and diffuse cutaneous SSc patients than in controls (*P* = 0.02 and *P* = 0.04, respectively). In SSc, decreased serum EGFL7 levels were significantly correlated with the severity of nailfold capillary abnormalities. Patients with the most severe capillary changes and digital ulcers had serum EGFL7 levels significantly lower than healthy controls, while the EGFL7 levels did not differ significantly between controls and SSc patients with less capillary damage and lack of digital ulcers. Endothelial EGFL7 expression was strongly downregulated or even almost completely undetectable in SSc-affected dermis compared with controls (*P* < 0.001). In cultured SSc dermal MVECs and late-outgrowth peripheral blood EPC-derived endothelial cells, EGFL7 was significantly downregulated compared with cells obtained from healthy subjects (*P* < 0.01 and *P* < 0.001, respectively).

**Conclusions:**

Our findings suggest that the loss of EGFL7 expression in endothelial cells and their progenitors might play a role in the development and progression of peripheral microvascular damage and the defective vascular repair process characteristic of SSc.

## Introduction

Systemic sclerosis (SSc, or scleroderma) is a chronic connective tissue disorder characterized by widespread microvascular injury, fibrosis and autoimmunity that affects the skin and internal organs [1]. In SSc, Raynaud’s phenomenon is the earliest clinical manifestation paralleled by nailfold capillaroscopic alterations that may occur months or even years before the onset of fibrosis. In the overall disease pathogenesis, this evidence suggests a crucial role of microvasculopathy characterized by a progressive and irreversible loss of capillaries and lack of compensatory angiogenesis/vasculogenesis often leading to severe peripheral ischemic manifestations [2,3].

Epidermal growth factor-like domain 7 (EGFL7), also known as vascular endothelial statin, is a recently discovered secreted protein that is expressed by and acts on endothelial cells and their mesodermal progenitors to control blood vessel development and integrity during both physiological and pathological vasculogenesis and angiogenesis [4,5]. Increasing evidence suggests that EGFL7 regulates blood vessel development by creating a permissive environment for angiogenesis [5]. The principal mechanism by which this occurs is by promoting endothelial cell proliferation, migration, sprouting and invasion [5-8]. Indeed, EGFL7 loss-of-function mouse models exhibit partial embryonic lethality and serious vascular abnormalities [7]. Moreover, EGFL7 plays a role in the repair of the microvasculature in response to vascular injury and ischemia [5,8]. Accordingly, increased levels of EGFL7 are detected in response to hypoxia in endothelial cells both *in vitro* and *in vivo* and stimulate an angiogenic response [9,10]. Finally, EGFL7 has a protective role against vascular injury by repressing key steps in the inflammatory activation of endothelial cells in response to hypoxia/reoxygenation [9,11].

On this basis, we hypothesized that EGFL7 might play a role in SSc pathogenesis. Therefore, the aim of the present study was to investigate whether the expression of EGFL7 could be altered in the circulation, skin and endothelial cells of SSc patients.

## Methods

### Patients, controls, serum samples and skin biopsy samples

Serum samples were obtained from 60 patients with SSc (56 women and 4 men; median age 56 years, range 24 to 73 years, and median disease duration 7 years, range 1 to 17 years), classified as limited cutaneous SSc (lcSSc; n = 37) or diffuse cutaneous SSc (dcSSc; n = 23) according to LeRoy *et al.*[12], and from 35 age- and sex-matched healthy individuals. All patients reported the occurrence of Raynaud’s phenomenon. At the time blood was drawn, the presence of digital ulcers was recorded. Nailfold videocapillaroscopy (NVC) was performed on all 10 fingers by a single rheumatologist and images were scored blindly to divide patients into three capillaroscopic patterns (that is, early, active and late) [13,14]. Table 1 shows the clinicodemographic characteristics of SSc patients used for collection of serum samples. Patients were not taking immunosuppressive medications, corticosteroids or other potentially disease-modifying drugs. Before blood sampling, they were washed out for 10 days from oral vasodilating drugs and for 2 months from intravenous prostanoids. Fresh venous blood samples were drawn, left to clot for 30 minutes before centrifugation at 1,500 *g* for 15 minutes, and serum was collected and stored in aliquots at -80°C until used. Paraffin-embedded sections of lesional forearm skin biopsies were obtained from 16 SSc patients (14 women and 2 men; n = 9 with lcSSc and n = 7 with dcSSc; median age 46.8 years, range 27 to 69 years, and median disease duration 6 years, range 1 to 16 years) and 10 age- and sex-matched healthy donors, as described elsewhere [14,15]. The study was approved by the local institutional review board at the Azienda Ospedaliero-Universitaria Careggi (AOUC), Florence, Italy, and all subjects provided written informed consent.

**Table 1:** Demographic and clinical characteristics of the 60 patients with SSc used for collection of serum samples

**Characteristic**	**Systemic sclerosis (SSc) patients**
Age, years, median (range)	56 (24 to 73)
Sex,	
Male	4 (6.7)
Female	56 (93.3)
Disease subset,	
lcSSc	37 (61.7)
dcSSc	23 (38.3)
Disease duration, years, median (range)^a^	7 (1 to 17)
Autoantibody positivity,	
ANA	56 (93.3)
Anti-Scl-70	20 (33.3)
ACA	25 (41.7)
Digital ulcers	25 (41.7)
Nailfold videocapillaroscopy pattern,	
Early	21 (35.0)
Active	24 (40.0)
Late	15 (25.0)
Skin score, median (range)^b^	10 (3 to 41)
Interstitial lung disease^c^	24 (40.0)

Except where indicated otherwise, values are the number (%) of subjects. ^a^Disease duration was calculated since the first non-Raynaud’s symptom of SSc; ^b^modified Rodnan skin thickness score; ^c^determined by high-resolution computer tomography. ACA, anticentromere antibodies; ANA, antinuclear antibodies; Anti-Scl-70, anti-Scl-70 antibodies; dcSSc, diffuse cutaneous SSc; lcSSc, limited cutaneous SSc.

### Isolation of dermal microvascular endothelial cells

Dermal microvascular endothelial cells (MVECs) were isolated from biopsy samples of the involved forearm skin from three patients with dcSSc and from three healthy subjects, as described elsewhere [14]. Patients were not taking immunosuppressive or disease-modifying drugs at the time of biopsy. Briefly, skin biopsy samples were mechanically cleaned of epidermis and adipose tissue in order to obtain a pure specimen of vascularized dermis, and were treated as previously described [14]. Colonies of polygonal elements were detached with ethylenediaminetetraacetic acid, and CD31-positive cells were subjected to immunomagnetic isolation. Isolated cells were further identified as MVECs by labeling with anti-factor VIII-related antigen and anti-CD105, followed by reprobing with anti-CD31 antibodies. MVECs were maintained in MCDB medium (Sigma-Aldrich, St Louis, MO, USA) supplemented with 30% fetal bovine serum, 20 μg/ml endothelial cell growth supplement (Calbiochem, Nottingham, UK), 10 μg/ml hydrocortisone, 15 IU/ml heparin, and antibiotics. MVECs were used between the third and seventh passages in culture.

### Late-outgrowth peripheral blood endothelial progenitor cell-derived endothelial cells

Late-outgrowth endothelial progenitor cell (EPC)-derived endothelial cells were obtained from the peripheral blood of 15 SSc patients (13 women and 2 men; n = 9 with lcSSc and n = 6 with dcSSc; median age 60 years, range 42 to 78 years) and 8 healthy individuals (all women; median age 55 years, range 30 to 65 years), as described elsewhere [16,17]. The study was approved by the local institutional review board at the Cochin Hospital, Paris, France, and all patients and control subjects provided written informed consent. Briefly, EPC isolation was performed on a 50-ml heparinized venous blood sample obtained from the forearm. Samples from hospitalized patients were obtained in the morning, at rest, during routine analysis. Patient and control samples were immediately transported to the laboratory for testing. The blood mononuclear cell fraction was collected by Ficoll density-gradient centrifugation and was resuspended in complete endothelial cell growth medium 2 (EGM-2; Lonza, Basel, Switzerland). Cells were then seeded onto separate wells of a 12-well tissue culture plate precoated with type I collagen (rat tail; BD Biosciences, Le Pont de Claix, France) and stored in an atmosphere of 5% CO_2_ at 37°C in a humidified incubator. After 24 hours of culture, non-adherent cells and debris were aspirated, adherent cells were washed once with PBS, and complete EGM-2 was added to each well. The medium was changed daily for 7 days and then every other day until the first passage. Colonies of endothelial cells appeared between 8 and 26 days of culture and were identified as well-circumscribed monolayers of cells with a cobblestone appearance. After the third passage, phenotyping of EPC-derived cells was performed by flow cytometry, as previously described [16]. After confirmation of the endothelial phenotype, cells were suspended in fetal bovine serum supplemented with 20% dimethyl sulfoxide, frozen in liquid nitrogen, and stored until used.

### ELISA for serum EGFL7

Serum EGFL7 levels were measured by colorimetric sandwich ELISA. First, 96-well microplates (R&D Systems, Minneapolis, MN, USA) were coated overnight at 4°C with rabbit polyclonal antihuman EGFL7 as a capture antibody (2.5 μg/ml; Abnova, Taipei, Taiwan). The wells were then washed twice with PBS with 0.1% Tween-20 and blocked for 2 hours at room temperature with 5% BSA in PBS with 0.1% Tween-20. Standards and serum samples (100 μl/well) were incubated for 2 hours at room temperature. The microplates were washed three times with PBS with 0.1% Tween-20, followed by the addition of mouse monoclonal antihuman EGFL7 as a detection antibody (1 μg/ml; Abnova) and horseradish peroxidase-conjugated goat anti-mouse immunoglobulin G (IgG). The reaction was developed with tetramethylbenzidine and then stopped by applying sulfuric acid (2 N H_2_SO_4_) (R&D Systems). The absorbance of each well was read using a microplate reader at 450 nm. Serum levels of EGFL7 were read from a standard curve prepared using recombinant human EGFL7 protein (Abcam, Cambridge, UK). The detection range of the assay was 0.3 to 30.0 ng/ml. Each sample was measured in duplicate.

### Immunofluorescence

For antigen retrieval, paraffin-embedded skin sections (5 μm thick) were deparaffinized and boiled for 10 minutes in sodium citrate buffer (10 mM, pH 6.0). The sections were washed three times in PBS, incubated in 2 mg/ml glycine for 10 minutes to quench autofluorescence caused by free aldehydes, and then blocked for 1 hour at room temperature with 1% BSA in PBS. The slides were incubated overnight at 4°C with mouse monoclonal antihuman EGFL7 antibody (catalogue number ab50254, Abcam) diluted 1:50 in PBS with 1% BSA. After extensive washing in PBS, the sections were incubated with Rhodamine Red-X-conjugated goat anti-mouse IgG (1:200 dilution; Molecular Probes, Eugene, OR, USA) for 45 minutes at room temperature in the dark. Irrelevant isotype-matched and concentration-matched mouse IgG (Sigma-Aldrich) were used as negative controls. For double immunofluorescence staining, we used a rabbit polyclonal antibody against CD31/pan-endothelial cell marker (1:50 dilution; catalogue number ab28364, Abcam) followed by Alexa Fluor-488-conjugated goat anti-rabbit IgG (1:200 dilution; Molecular Probes). Nuclei were counterstained with 4′,6-diamidino-2-phenylindole (DAPI) (Chemicon International, Temecula, CA, USA). The immunolabeled sections were then observed under a Leica DM4000 B microscope equipped with fully automated fluorescence axes (Leica Microsystems, Mannheim, Germany). Fluorescence images were captured using a Leica DFC310 FX 1.4-megapixel digital colour camera equipped with the Leica software application suite LAS V3.8 (Leica Microsystems). Densitometric analysis of the intensity of immunofluorescent staining was performed on digitized images using the free-share ImageJ software (NIH, Bethesda, MD, USA; online at http://rsbweb.nih.gov/ij).

### Western blotting

Proteins were extracted from dermal MVECs and late-outgrowth peripheral blood EPC-derived endothelial cells as described elsewhere [14,17]. Twenty micrograms of total proteins were electrophoresed on NuPAGE 4 to 12% Bis-Tris Gel (Invitrogen, Carlsbad, CA, USA) and blotted onto polyvinylidene difluoride membranes (Invitrogen). The membranes were blocked with blocking solution included in the Western Breeze Chromogenic Western Blot Immunodetection Kit (Invitrogen) for 30 minutes at room temperature on a rotary shaker and incubated for 1 hour at room temperature with mouse monoclonal antihuman EGFL7 (1:500 dilution; Abcam) and rabbit polyclonal anti-α-tubulin (catalogue number #2144; 1:1,000 dilution; Cell Signaling Technology, Danvers, MA, USA) antibodies, assuming α-tubulin as control invariant protein. Immunodetection was performed as described in the Western Breeze Chromogenic Immunodetection protocol (Invitrogen). Densitometric analysis of the bands was performed using ImageJ software and the values normalized to α-tubulin.

### Statistical analysis

Data are expressed as the mean ± standard error of the mean (SEM) or median and IQR. The Student *t*-test and nonparametric Mann–Whitney *U*-test were used where appropriate for statistical evaluation of the differences between two independent groups. A *P*-value less than 0.05 was considered statistically significant.

## Results

**Figure 1:**
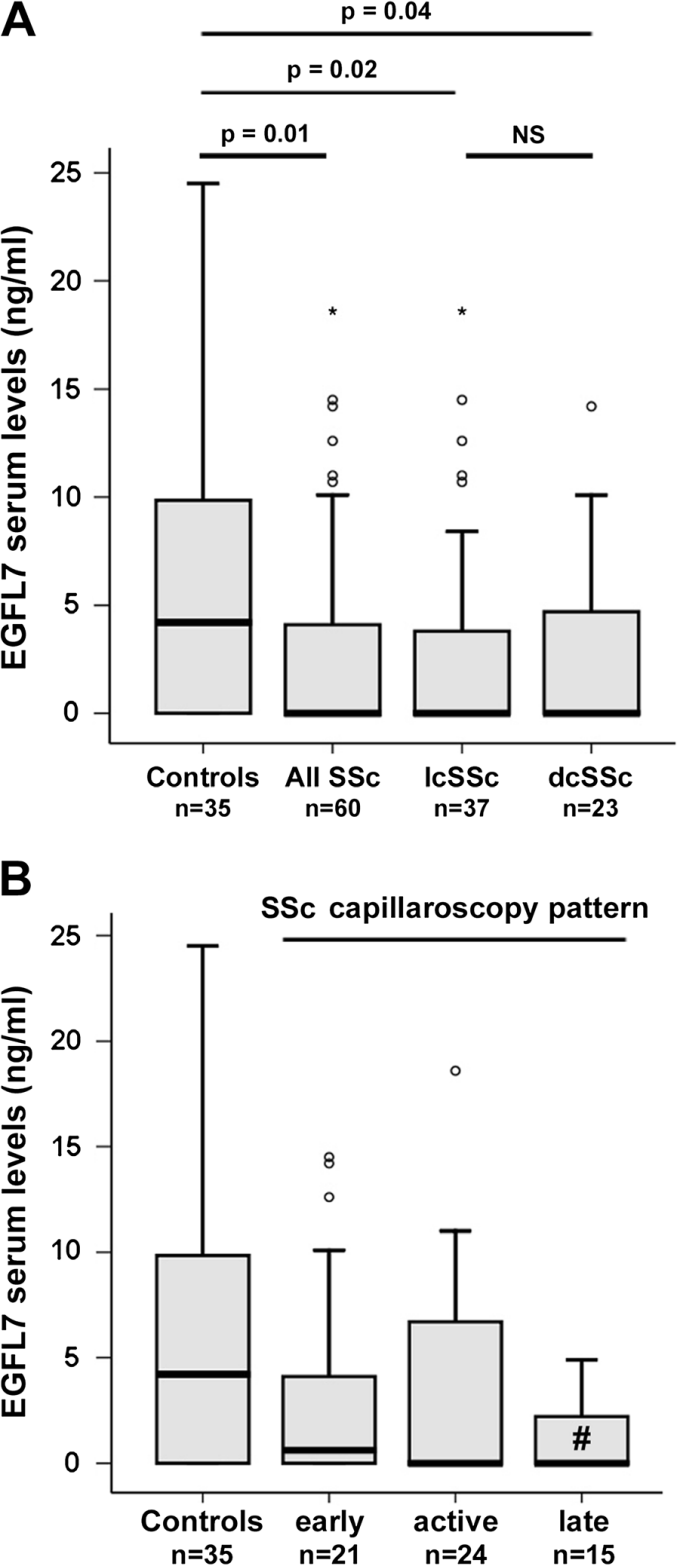
**Serum levels of epidermal growth factor-like domain 7 (EGFL7) determined by colorimetric sandwich ELISA. (A)** Serum EGFL7 levels in healthy controls, all patients with systemic sclerosis (SSc), patients with limited cutaneous SSc (lcSSc) and patients with diffuse cutaneous SSc (dcSSc). **(B)** Serum levels of EGFL7 in patients with SSc according to nailfold videocapillaroscopy pattern (early, active and late). Data are shown as box plots. Each box represents the 25th to 75th percentiles. Lines inside the boxes represent the median. Lines outside the boxes represent the 10th and the 90th percentiles. Circles indicate outliers, and asterisks indicate the extreme values. ^#^*P* = 0.006 versus controls. Mann–Whitney *U*-test was used for statistical analysis. NS, not significant.

Serum EGFL7 levels were detectable in 68.6% of healthy subjects and 45% of SSc patients (*P* <0.05, χ^2^ test). Circulating levels of EGFL7 were significantly decreased in SSc patients (median 0.0 ng/ml, IQR 0.0 to 4.1 ng/ml) compared with healthy controls (median 4.2 ng/ml, IQR 0.0 to 10.3 ng/ml; *P* = 0.01) (Figure 1A). Serum EGFL7 levels were significantly lower both in lcSSc patients (median 0.0 ng/ml, IQR 0.0 to 3.9 ng/ml) and dcSSc patients (median 0.0 ng/ml, IQR 0.0 to 5.3 ng/ml) than in controls (*P* = 0.02 and *P* = 0.04, respectively) (Figure 1A). No significant difference in circulating EGFL7 protein levels was detected between lcSSc and dcSSc (Figure 1A).

Next, we evaluated the possible correlation of serum EGFL7 levels with the NVC pattern as a measure of peripheral microvascular involvement. EGFL7 levels were lower in SSc patients with late NVC pattern (median 0.0 ng/ml, IQR 0.0 to 2.7 ng/ml) than in those with early (median 0.6 ng/ml, IQR 0.0 to 6.2 ng/ml) or active (median 0.0 ng/ml, IQR 0.0 to 7.1 ng/ml) patterns, but these differences did not reach statistical significance (Figure 1B). Serum EGFL7 levels were significantly decreased in SSc patients with late NVC pattern compared with healthy controls (*P* = 0.006), whereas although decreased, no significant difference in EGFL7 levels could be found between SSc patients with early or active NVC patterns and controls (Figure 1B). A trend toward reduction in the circulating levels of EGFL7 was observed in SSc patients with digital ulcers (median 0.0 ng/ml, IQR 0.0 to 2.2 ng/ml) compared with those without digital ulcers (median 0.6 ng/ml, IQR 0.0 to 8.0 ng/ml; *P* = 0.08). Moreover, EGFL7 levels were significantly lower in SSc patients with digital ulcers than in controls (*P* = 0.002), whereas no difference was detected when comparing the group of SSc patients without digital ulcers to controls. No significant association was found with the other clinicodemographic and laboratory parameters.

**Figure 2:**
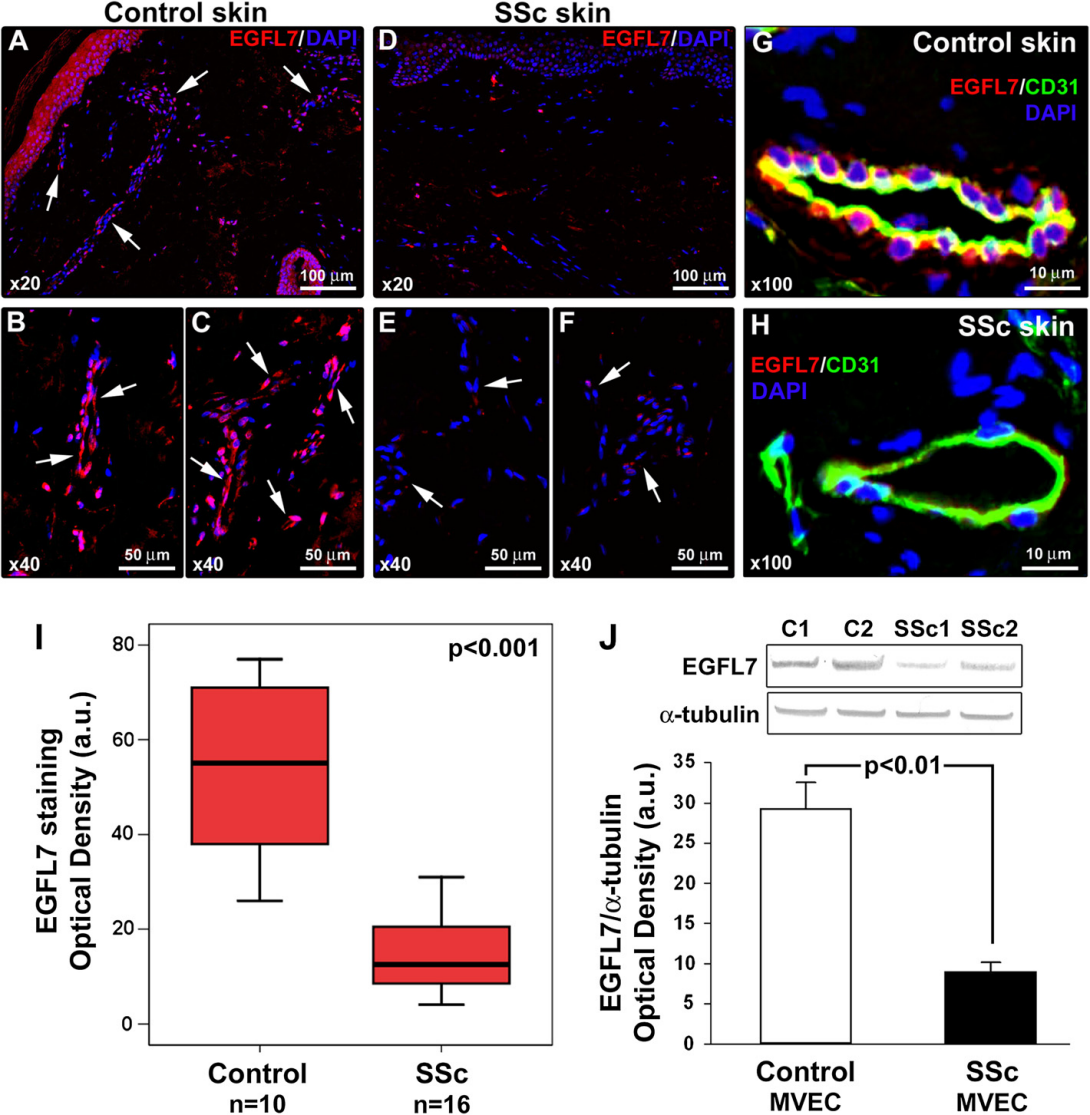
**Decreased expression of epidermal growth factor-like domain 7 (EGFL7) in the affected skin and dermal microvascular endothelial cells (MVEC) of patients with systemic sclerosis (SSc). (A-F)** Representative microphotographs of skin sections from healthy controls (**A**-**C**; n = 10) and SSc patients (**D**-**F**; n = 16) immunostained for EGFL7 (red) and counterstained with 4′,6-diamidino-2-phenylindole (DAPI; blue) for nuclei. Arrows indicate microvessels. **(G and H)** Representative microphotographs of skin sections from healthy controls **(G)** and SSc patients **(H)** double immunostained for EGFL7 (red) and the pan-endothelial cell marker CD31 (green) and counterstained with DAPI (blue). Original magnification and scale bars are indicated in each panel. **(I)** Densitometric analysis of EGFL7 immunofluorescent staining in dermal microvessels expressed as optical density in arbitrary units (a.u.). Data are shown as box plots. Each box represents the 25th to 75th percentiles. Lines inside the boxes represent the median. Lines outside the boxes represent the 10th and the 90th percentiles. Mann–Whitney *U*-test was used for statistical analysis. **(J)** Western blotting of protein lysates from control dermal MVEC (n = 3) and SSc MVEC (n = 3) analyzed using anti-EGFL7 antibodies. Representative immunoblots are shown. The densitometric analysis of the bands normalized to α-tubulin is reported in the histograms. Data are mean ± standard error of the mean of optical density in a.u. Student *t*-test was used for statistical analysis.

**Figure 3:**
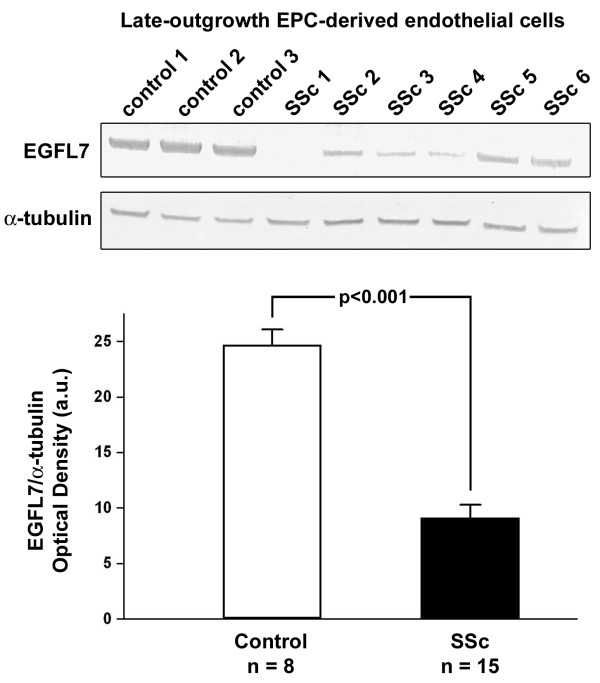
**Decreased expression of epidermal growth factor-like domain 7 (EGFL7) in late-outgrowth peripheral blood endothelial progenitor cell (EPC)-derived endothelial cells from patients with systemic sclerosis (SSc).** Western blotting of protein lysates from control (n = 8) and SSc (n = 15) late-outgrowth peripheral blood EPC-derived endothelial cells analyzed using anti-EGFL7 antibodies. Representative immunoblots are shown. The densitometric analysis of the bands normalized to α-tubulin is reported in the histograms. Data are mean ± standard error of the mean of optical density in arbitrary units (a.u.). Student *t*-test was used for statistical analysis.

The expression of EGFL7 protein in forearm skin biopsies from SSc patients and controls was investigated by immunofluorescence. Constitutive EGFL7 expression was observed in dermal endothelial cells from healthy control skin (Figure 2A-C). Endothelial EGFL7 expression was strongly downregulated or even almost undetectable in SSc-affected dermis (Figure 2D-F). The localization of EGFL7 staining in vascular endothelial cells was confirmed by EGFL7/CD31 double immunofluorescence staining (Figure 2G,H). Densitometric analysis of immunofluorescent staining intensity on skin sections showed that EGFL7 protein expression was significantly decreased in dermal vessels of SSc patients compared with controls (*P* <0.001) (Figure 2I). No significant differences in dermal expression of EGFL7 were observed according to disease duration or cutaneous SSc subsets. Moreover, Western blot analysis revealed that EGFL7 protein expression levels were significantly reduced in MVECs isolated from dcSSc dermis compared with control dermal MVECs (*P* <0.01) (Figure 2J). Consistent with skin and dermal MVEC findings, EGFL7 protein levels were significantly lower in late-outgrowth peripheral blood EPC-derived endothelial cells from SSc patients than in cells obtained from healthy controls (*P* <0.001; Figure 3). No obvious differences in EGFL7 levels could be found between late-outgrowth EPC-derived endothelial cells from lcSSc and dcSSc patients.

## Discussion

This is the first study to investigate the possible involvement of the recently identified angiogenic signaling molecule EGFL7 in the pathogenesis of SSc. Our data show that serum levels and dermal expression of EGFL7 are significantly decreased in SSc patients. Remarkably, the decrease of serum EGFL7 levels in SSc patients correlated with the severity of nailfold capillary abnormalities. In contrast to constitutive endothelial cell expression of EGFL7 in healthy skin, EGFL7 was found to be strongly reduced or even undetectable in SSc dermal microvessels. Furthermore, EGFL7 protein was found to be significantly downregulated *in vitro* both in dermal MVECs and peripheral blood EPC-derived endothelial cells obtained from SSc patients compared with the respective control cells. Taken together, our findings suggest that the loss of EGFL7 expression might play a role in the development and progression of peripheral microvascular damage and defective vascular repair process in SSc patients.

The majority of secreted angiogenic signaling molecules are expressed not only by endothelial cells but also by a plethora of other cell types, such as macrophages, fibroblasts, smooth muscle cells and epithelial cells [5]. In this context, EGFL7 is unique because it is almost exclusively expressed by and acts on endothelial cells [5-8,18]. During embryogenesis, EGFL7 expression is detected at sites of both extra-embryonic and embryonic mesodermal progenitors and in vascular structures that arise by vasculogenesis [4-6]. Most adult tissues express EGFL7 in a subset of vessels, however, the levels are considerably lower in quiescent endothelium than in the embryo [6,8]. EGFL7 expression is elevated in the proliferating endothelium of the pregnant uterus, and increased levels are observed during tumorigenesis or endothelial regeneration subsequent to vascular injury, suggesting that it plays a role during both physiological and pathological angiogenesis and vascular remodeling [5,6,8,18]. Indeed, EGFL7 regulates multiple steps in the angiogenic cascade, including endothelial cell proliferation, migration, adhesion, sprouting and invasion [5-8,19]. In particular, it has been recently demonstrated that EGFL7 promotes angiogenesis mainly via its interaction with the integrin αvβ3 receptor [20]. EGFL7 is secreted by endothelial cells into the extracellular matrix, where it affects the process of lumen formation [21]. Furthermore, miRNA-126, a microRNA located within the *EGFL7* gene, may also have a major role in vessel development by promoting vascular endothelial growth factor signaling, angiogenesis and vascular integrity [21]. Recent studies suggest a protective role for EGFL7 against vascular injury and ischemia. In support, EGFL7 expression is temporarily induced in regenerating endothelium of mice that have been subjected to arterial vascular insult [8]. Interestingly, an increase in the levels of EGFL7 under hypoxic conditions appears not only to stimulate compensatory angiogenesis, but also to repress key steps in the inflammatory activation of endothelial cells in response to ischemia/reperfusion injury [5,9,10].

In this context, the correlation of serum EGFL7 levels with the severity of SSc-related peripheral microvascular damage appears to be of major importance. In fact, serum EGFL7 levels progressively decreased reaching the lowest values in SSc patients with the late NVC pattern, which is characterized by substantial loss of capillaries with formation of avascular areas [13]. Moreover, patients with most severe capillary changes and digital ulcers had serum EGFL7 levels significantly lower than healthy controls, whereas the EGFL7 levels did not differ significantly between controls and SSc patients with less capillary damage and lack of digital ulcers. Despite the hypoxic microenvironment of SSc skin [3,22], the expression of EGFL7 was strongly downregulated *ex vivo* in dermal microvessels from lesional forearm skin biopsies. Finally, EGFL7 downregulation was consistently observed *in vitro* not only in SSc dermal MVECs, but also in peripheral blood EPC-derived endothelial cells, thus indicating that EGFL7 signaling defects may even originate in committed endothelial lineage progenitors. Collectively, these findings suggest that failure to induce EGFL7 expression following endothelial cell activation/damage might have a role in the defective vascular repair process of SSc. However, since EGFL7 is specifically released by endothelial cells, we should also consider that the reduction in circulating levels of EGFL7 might be either a cause or a consequence of the disease, which is characterized by progressive loss of the peripheral microcirculation [3]. Serum levels of EGFL7 could even serve as a biomarker reflecting the severity of microvascular involvement in SSc.

## Conclusions

In summary, we shed light on EGFL7 as a possible new player in the vascular component of SSc pathogenesis. Further functional studies are now required to ascertain whether restoration of endothelial EGFL7 expression might offer new targeted therapeutic strategies to control the progression of peripheral microvasculopathy in SSc.

## Abbreviations

a.u.: Arbitrary units; BSA: Bovine serum albumin; DAPI: 4′,6-diamidino-2-phenylindole; dcSSc: Diffuse cutaneous SSc; EGFL7: Epidermal growth factor-like domain 7; EGM: Endothelial cell growth medium; ELISA: Enzyme-linked immunosorbent assay; EPC: Endothelial progenitor cell; IgG: Immunoglobulin G; lcSSc: Limited cutaneous SSc; MVECs: Microvascular endothelial cells; NVC: Nailfold videocapillaroscopy; PBS: Phosphate-buffered saline; SEM: Standard error of the mean; SSc: Systemic sclerosis.

## Competing interests

The authors declare that they have no competing interests.

## Authors’ contributions

All authors meet the criteria for authorship. MM conceived the study, participated in its design and coordination, contributed to most of the experiments, analysis and interpretation of data, and drafted and edited the manuscript. SG participated in study design, dermal MVEC isolation and culture, and collected and supplied biological samples and clinical data. ER contributed to dermal MVEC isolation and culture, ELISA, Western blotting and analysis and interpretation of data. JA and BR performed isolation and culture of peripheral blood EPC-derived endothelial cells and supplied clinical data. IR contributed to the immunofluorescence experiments and analysis of data. GL and SB-R collected and supplied serum samples and clinical data. LI-M, YA and MM-C participated in study design and coordination and interpretation of data, drafted the article and revised it critically for important intellectual content. All authors approved the final version of the manuscript to be published.
